# Implementing Interventions Under “National Action Plan for Snakebite Envenoming (NAPSE) in India”: Challenges, Lessons Learnt and Way Forward for Stakeholders Participatory Approach

**DOI:** 10.3390/tropicalmed10050132

**Published:** 2025-05-14

**Authors:** Ajit Dadaji Shewale, Dipti Mishra, Simmi Tiwari, Tushar Nanasaheb Nale, Jitesh Kuwatada, Nidhi Khandelwal

**Affiliations:** Centre for One Health, National Centre for Disease Control, Ministry of Health & Family Welfare, New Delhi 110074, India; dr.diptimishra@gmail.com (D.M.); drsimmi@ncdc.gov.in (S.T.); tushar.nale@nic.in (T.N.N.); jitesh.kuwatada@ncdc.gov.in (J.K.); knidhi22@gmail.com (N.K.)

**Keywords:** snakebite envenoming, public health, anti-snake venom, India, NAPSE, healthcare infrastructure, One Health approach, surveillance systems, rural health, community awareness

## Abstract

Snakebite envenoming remains a critical yet underrecognized public health issue, particularly in tropical and subtropical regions, with India bearing nearly half of the global burden of snakebite-related deaths. Despite its significant impact, underreporting, delayed medical intervention, and insufficiently trained healthcare professionals continue to exacerbate the problem. In response, the Government of India launched the National Action Plan for Prevention and Control of Snakebite Envenoming (NAPSE) in March 2024, aiming to halve snakebite-related deaths by 2030. Key challenges during the development and implementation of NAPSE included the limited multisectoral engagement initially, variations in state-level capacities, and logistical barriers in reaching remote populations. Lessons learned include the value of early stakeholder consultations, the importance of inter-ministerial collaboration, and the need for continuous community engagement. This comprehensive strategy emphasizes strengthening surveillance systems, enhancing anti-snake venom (ASV) distribution and quality, improving healthcare infrastructure, and promoting community awareness through a One Health approach. The plan also addresses critical challenges such as inadequate training at primary healthcare levels, inconsistent ASV supply, and inefficient emergency referral systems. By fostering multisectoral collaboration and targeted interventions, such as strengthening Regional Venom Centres and establishing Poison Information Centre, targeted training, and awareness campaigns, NAPSE aims to reduce mortality and disability associated with snakebite envenoming, aligning with global health objectives and setting an example for regional efforts in Southeast Asia.

## 1. Introduction

Snakebite envenoming (SBE) is a significant public health concern, particularly in tropical and subtropical regions [1]. As per the World Health Organization (WHO), approximately five million people are bitten by snakes, and every year, 1.8 to 2.7 million people suffer from Envenoming [1]. It leads to disabilities and psychological consequences in the aftermath of snakebites, particularly in developing regions. Underprivileged and rural communities in tropical and subtropical countries face major consequences [2].

Considering the impact, WHO listed snakebite envenoming as a priority neglected tropical disease in 2017. It was recognized as developing and tropical countries facing almost 95% of the world’s snakebite-related burden. The decision aimed to highlight the urgency and create opportunities for increased investment in research funding to improve snakebite management in these regions. One of the primary reasons for its earlier removal from the Neglected Tropical Diseases (NTD) list was the lack of sufficient data from developing countries like India, which made it challenging to fully grasp the extent of the problem [3].

SBE continues to be a significant yet long-neglected public health challenge in India, disproportionately affecting rural and tribal populations, particularly agricultural and forest-based communities [4]. India accounts for nearly half of the global snakebite deaths, with an estimated 58,000 annual fatalities and three times as many long-term disabilities [1].

India’s response to this public health challenge has evolved over time. The journey began with anti-venom (ASV) manufacturing at the Central Research Institute in Kasauli in 1920, followed by the Haffkine’s Institute in Mumbai in 1945 [2]. Earlier approaches primarily focused on clinical management through training medical officers and ensuring the availability of anti-snake venom (ASV) in public health facilities. These efforts included the release of the Standard Treatment Guidelines for Snakebite Management in 2017, which was further refined in 2019 by the Ministry of Health and Family Welfare (MoHFW), which served as a guiding document for clinicians [5].

Further, the Indian Council of Medical Research (ICMR) established a National Task Force Expert Group on Research on Snakebite in India. In 2019, two major research studies were funded under this initiative—one aimed at comprehensive data discovery to assess the burden, and the other focused on implementation research for health system strengthening [5].

The evidence generated through these efforts has brought to light stark regional disparities in snakebite incidence, mortality, morbidity, and the capacity of the health system to respond. These findings emphasize that enhancing health infrastructure, empowering communities with accurate knowledge, and tailoring interventions to local contexts are pivotal for reducing the snakebite burden.

Further, a white paper was released by ICMR in 2020 [3], which discussed the various reasons that could be attributed to the high mortality and morbidity due to venomous snakes and provided recommendations on policy decisions, improvement on the quality of venom and anti-snake venom and in promoting awareness on how to avoid snakebite. A Nationally Representative Mortality Survey conducted in March 2001 [6] in India has highlighted that snakebite remains an underestimated cause of accidental death in modern India, and it is significantly underreported. The study suggested that an effective intervention involving education and anti-venom provision would reduce snakebite deaths in India.

The absence of a coordinated, multisectoral, and evidence-based approach has hindered the effectiveness of these interventions. Recognizing the urgent need for a structured, integrated, and sustainable approach, the National Action Plan for Prevention and Control of Snakebite Envenoming in India was developed. This plan aims to provide a comprehensive framework for reducing snakebite-related deaths and disabilities through coordinated actions across health, veterinary, environmental, and community sectors.

## 2. Current Scenario in India

Earlier research studies in India have shown that snakebite accounts for nearly half of the world’s snakebite-related deaths, with approximately 58,000 deaths resulting from an estimated 3–4 million snakebites annually [7]. It is highlighted in these studies that a significant number of snakebite victims do not seek medical attention at clinics or hospitals, leading to a substantial underreporting of cases [2]. The burden of premature deaths due to snakebite envenoming in India is estimated at 2.97 million disability-adjusted life years (DALYs), compared to the global burden of 6.07 million DALYs [8].

Currently, snakebite cases and related deaths in India are monitored through the Integrated Diseases Surveillance Program–Integrated Health Information Portal (IDSP–IHIP). Community health workers document snakebite cases using the S (Suspected) form, while Medical Officers (MO) report presumptive snakebite cases and fatalities using the P form. Recently, IDSP launched a community reporting tool for any outbreak-related alert. In 2024, over 1.20 Lakhs cases were reported, with 370 deaths.

This gap in reporting is being addressed through continued IHIP training across all states and sensitization by the program division to gather snakebite-related data from stakeholders at the field level, i.e., forest department, agriculture department, and revenue department mainly documented for compensation/medico-legal requirements.

A dedicated informational and data portal is also being developed in the IHIP portal as part of the recent notification of snakebite cases and deaths, which in turn is expected to improve reporting from all sectors, including the private sector.

**Figure 1:**
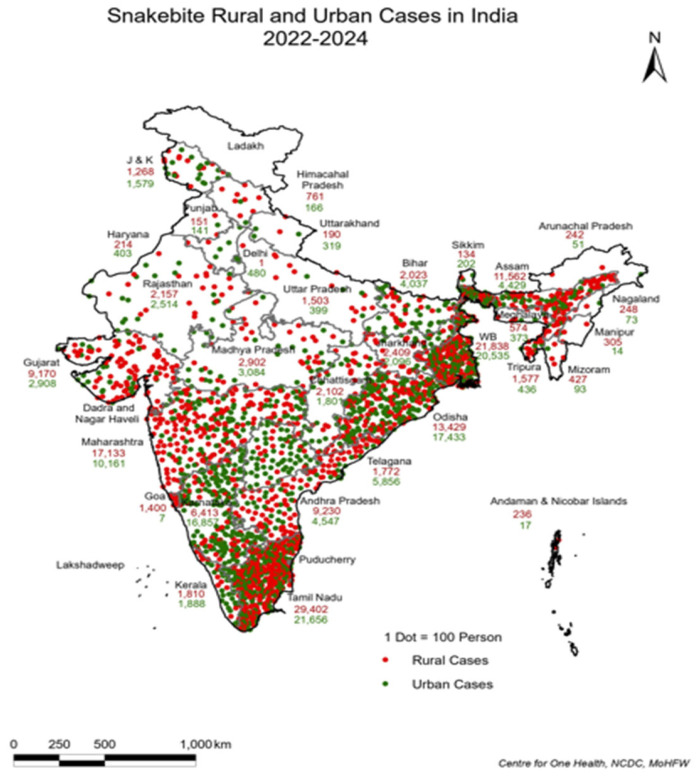
Reported Snakebite Cases in India (Source–IDSP/IHIP).

This staggering burden, primarily concentrated in rural (Figure 1) and agricultural communities, is exacerbated by factors such as underreporting cases, delayed access to treatment, and a lack of adequately trained healthcare professionals. The bites of the “big four” venomous snake species, i.e., Indian cobra (*Naja naja*), Russell’s viper (*Daboia russelii*), common krait (*Bungarus caeruleus*), and saw-scaled viper (*Echis carinatus*) account for most of these cases.

As per the reported number of snakebites in IDSP, 46% of cases are being reported in rural areas. However, Figure 1 also illustrates that snakebites are now increasingly reported from urban areas, likely due to unplanned urbanization, climate change, water logging, etc.

**Figure 2:**
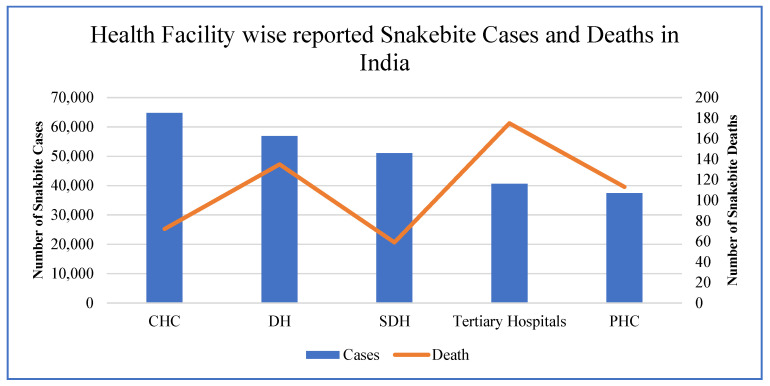
Health Facilities-Reported Snakebite Cases and Deaths from 2020–2024 (Source–IDSP/IHIP).

Figure 2 illustrates the distribution of snakebite cases and related deaths across different health facilities in India, highlighting significant variations in case reporting and fatality rates. Community Health Centers (CHCs) report the highest number of cases, followed by District Hospitals (DHs) and Sub-District Hospitals (SDHs), while Primary Health Centers (PHCs) record the lowest. However, mortality trends show a contrasting pattern, with tertiary hospitals experiencing the highest number of deaths, likely due to delayed referrals and patients arriving in critical condition.

This is also corroborated by research studies undertaken in India, e.g., Mohapatra et al. (2011) [9] indicated that CHCs and DHs are the primary points of care for snakebite victims in rural India, whereas higher fatalities at tertiary hospitals suggest inefficiencies in early intervention. Additionally, PHCs report fewer cases but relatively higher mortality, underscoring the lack of adequate treatment facilities at the primary level. Other research studies also emphasize the same findings with an urgent need for improved snakebite management, timely referrals, and better-equipped rural healthcare centers to reduce mortality rates. Accordingly, under the Health System Strengthening (HSS) component of the National Health Mission (NHM), issues are being addressed through improved and timely transport of patients as part of 108 ambulance services. This ensures the availability of ASV and other emergency drugs, e.g., neostigmine up to the PHC level, and upskilling of medical officers at the PHC level to manage these cases.

**Figure 3:**
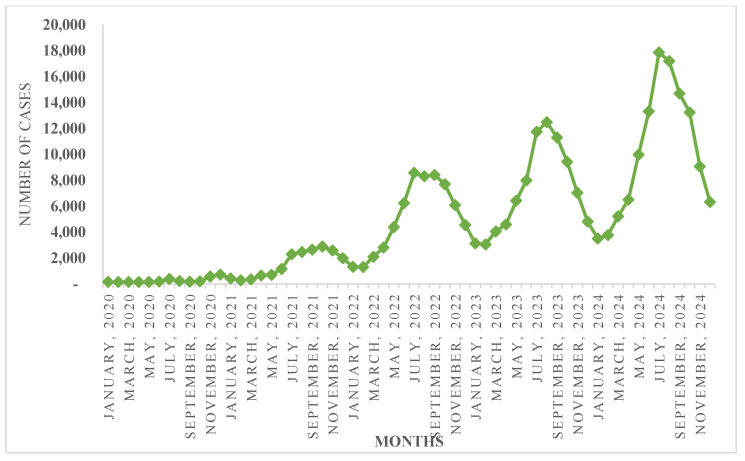
Seasonality under Snakebite Cases (Reported from 2020–2024 as per IDSP/IHIP).

Data from IDSP–IHIP also reveals that snakebite cases typically peak in June and start declining in October, showing a seasonal pattern of increased incidents over the years (Figure 3). Densely populated, low-altitude agricultural regions in states such as Karnataka, West Bengal, Jharkhand, Odisha, and Madhya Pradesh account for the majority of snakebite deaths, particularly during the rainy season when human–snake encounters are more common both indoors and outdoors. Considering these trends, a checklist for ensuring the availability of logistics and equipment is prepared, and states are encouraged to undertake training as part of pre-monsoon preparedness.

Addressing the immense burden of snakebite envenoming in India requires concerted and collaborative efforts to strengthen surveillance, enhance access to timely medical care, and promote community awareness.

## 3. Journey Towards Development of National Action Plan for Prevention and Control of Snakebite Envenoming (NAPSE) in India

Snakebite envenoming remains a critical yet often overlooked public health concern in India, particularly in rural and underserved regions where timely medical intervention is limited. To address this pressing issue, the Government of India initiated the development of the National Action Plan for Prevention and Control of Snakebite Envenoming in India (NAPSE), a comprehensive framework built through extensive collaboration, expert consultations, and community engagement.

The foundation for NAPSE was laid during a regional consultation on snakebite envenoming in the Southeast Asia Region (SEAR) in March 2022, where experts emphasized the need for a structured and strategic plan to tackle the multifaceted challenges of snakebite envenoming, especially among vulnerable populations.

During the consultation, it was highlighted that only 30% of SEAR countries have a national program dedicated to the prevention and control of snakebite envenoming. Among these, Myanmar, Nepal, and Thailand have established programs under respective health authorities.

A concerning finding was that only two countries, Bangladesh and Thailand, had a dedicated national strategy or plan for addressing snakebite envenoming.

Recognizing the urgency, the first National Consultation on Developing an Action Plan for Prevention and Control of Snakebite Envenoming was convened on 26 July 2022 under the leadership of the Directorate General of Health Services (DGHS), Ministry of Health and Family Welfare. This event marked a pivotal step in solidifying India’s commitment to addressing snakebite envenoming at a national level.

A major outcome of the consultation was the recommendation to establish a dedicated program under the National Health Mission (NHM) to ensure uniform implementation of snakebite prevention and control initiatives across states and union territories. In response, the Centre for One Health, under the National Centre for Disease Control (NCDC), developed a proposal advocating for the inclusion of snakebite prevention activities under NHM. This proposal was subsequently presented at the 9th EPC meeting on 18 August 2022 and received approval from the Mission Steering Group of NHM, marking a significant milestone in integrating snakebite envenoming into India’s national health agenda.

As per the recommendation of the consultation, a core committee was constituted to draft NAPSE. Between February and March 2023, multiple consultative meetings were held with key stakeholders, including anti-snake venom manufacturers, NGOs, clinical experts, and communication officers.

**Figure 4:**
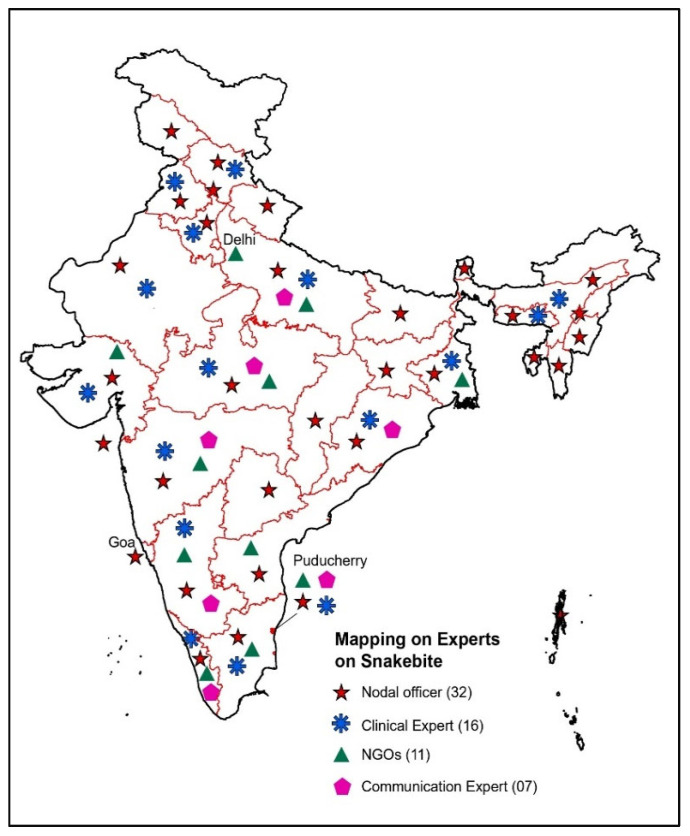
Mapping of Key Experts in States/UTs.

**Figure 5:**
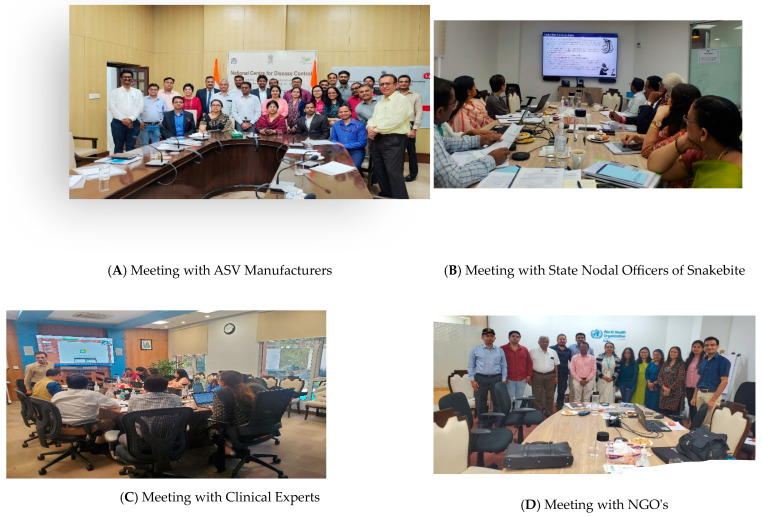
Snapshots of Meeting with Experts. (*Photo owner—Programme division*).

The Program division has mapped experts such as State Nodal Officers, Clinical Experts, NGOs, Communication experts, etc., in all States/UTs (Figure 4 and Figure 5). Their collective insights ensured that the plan was evidence-based and tailored to real-world challenges.

An inter-ministerial meeting on 28 August 2023 further reinforced a collaborative approach by bringing together various government ministries to define their roles and responsibilities under NAPSE. This initiative aimed to facilitate seamless implementation and align efforts across multiple sectors. Furthermore, the drafting process involved direct engagement with communities and healthcare professionals to identify on-ground challenges and ensure that the plan addressed the specific needs of those most affected by snakebites.

Understanding the importance of grassroots involvement, the drafting process also included community interactions and consultations with clinicians to identify challenges and requirements from the ground level. This ensured that the plan was not only comprehensive but also tailored to address the specific needs of communities most affected by snakebites.

The culmination of these efforts was the official launch of the National Action Plan for Prevention and Control of Snakebite Envenoming in India (NAPSE) in March 2024. The plan is a landmark initiative that aims to halve snakebite-related deaths by 2030, reflecting the government’s commitment to saving lives and improving public health. NAPSE adopts a One Health approach, integrating human, animal, and environmental health strategies to address snakebite envenoming comprehensively.

The key milestones in the planning and development of NAPSE are described in Table 1.

It focuses on key areas such as strengthening the production and distribution of high-quality anti-venom, building healthcare capacity, enhancing transportation and emergency response systems, raising community awareness, and fostering multisectoral collaboration.

With this, India aims to lead the efforts in the Southeast Asia region for snakebite prevention and control efforts and contribute to the global goal of WHO to halve deaths by 2030.

A summary of initiatives by different countries in this regard is described in Table 2.

## 4. Challenges in the Implementation of NAPSE

The implementation of the National Action Plan for Prevention and Control of Snakebite Envenoming (NAPSE) faces several challenges that could hinder its implementation. One of the most significant issues is the underreporting of snakebite cases due to inadequate surveillance systems and cultural stigma, which limits the availability of accurate data for informed decision-making. Ensuring consistent access to quality anti-snake venom (ASV) in rural and remote areas remains a logistical challenge. Many healthcare professionals, especially in primary health care, lack adequate training in managing snakebite envenoming, leading to delays or mismanagement in treatment. Poor emergency referral systems, including the lack of ambulances, further exacerbate the problem. Public awareness is another critical gap, as myths and misconceptions about snakebites often drive victims to seek harmful traditional remedies instead of timely medical care.

The success of NAPSE also depends on effective inter-sectoral coordination, which is often hampered by overlapping mandates and poor communication among stakeholders in the human health, wildlife, and veterinary sectors. Moreover, behavioral resistance among communities to adopt preventive measures or seek medical care promptly remains a persistent challenge. Addressing these challenges will require strengthening surveillance systems, ensuring equitable ASV distribution, enhancing healthcare provider training, establishing robust referral mechanisms, fostering inter-sectoral collaboration, and launching sustained awareness campaigns tailored to regional needs. By overcoming these barriers, NAPSE can achieve its mission of reducing snakebite-related deaths and disabilities across India.

## 5. Way Forward

Research studies over the years from 2000–2019 have identified several critical gaps in snakebite management across venom supply, legislation, medical care, and public awareness. Venom-related gaps include the absence of zonal venom banks, exclusion of region-specific venoms in ASV manufacturing, reliance on wild snakes for venom collection, lack of quality testing, unregulated venom pricing, and insufficient research on alternative ASV production techniques and monovalent ASVs. Legislative gaps involve the lack of mandatory reporting, standardized ICD coding, school-based awareness programs, and the medico-legal classification of snakebites, which delays treatment. Medical and diagnostic gaps include inadequate healthcare worker training, absence of venom detection kits, non-uniform treatment protocols, lack of curriculum integration, and exclusion of snakebite treatment from government health schemes. Public awareness and outreach gaps stem from limited prevention knowledge, the absence of large-scale preventive measures, continued belief in traditional remedies, and the lack of a 24/7 snakebite helpline.

To address the numerous challenges and achieve the objectives of the National Action Plan for Snakebite Envenoming (NAPSE), a well-rounded, multifaceted approach is essential. Recognizing the complexity of the issue and the diverse needs across the country, the ministry has devised a strategic roadmap to ensure no region or community is left behind in the fight against snakebite mortality and morbidity.

A critical step in this direction is the designation of snakebite as a notifiable disease in India, strengthening surveillance and data collection efforts. This measure will enhance the ability to track cases accurately, identify high-risk areas, and allocate resources effectively for prevention and treatment.

Currently, the priority is to improve the safety and quality of current polyvalent snake venom. A committee on monitoring antisera has been constituted to review the above issues with manufacturers and regulators. Collaboration is being made with domestic and international stakeholders to support the venom collection centers for improving the quality of venom. Further, one of the important initiatives under the program is to strengthen the Regional Venom Centers in five regions of India. These Regional Venom Centers will focus on conducting region-specific venom research on the Big Four snake species—Indian cobra (*Naja naja*), Russell’s viper (*Daboia russelii*), common krait (*Bungarus caeruleus*), and saw-scaled viper (*Echis carinatus*)—as well as other venomous species unique to different zones. A key priority will be the development of region-specific ASVs, ensuring that anti-venom formulations effectively neutralize the venoms of species prevalent in specific geographical areas. Studies suggest that region-specific anti-snake venoms (ASVs) significantly improve treatment outcomes compared to generalized ASVs. Research studies [3] show that tailored ASVs can enhance neutralization efficiency and reduce treatment-related complications. By strengthening these research and production capabilities, the Regional Venom Centers will play a crucial role in improving snakebite treatment, reducing mortality, and enhancing public health outcomes across India.

In addition to improving access to anti-venom, strengthening Poison Information Centers (PICs) in every state and union territory is an important initiative under the program. These centers will serve as knowledge hubs, equipping healthcare professionals with evidence-based guidelines, training, and support for snakebite management. Standardizing treatment protocols through PICs ensures that snakebite victims receive prompt and effective care, regardless of their location. The current protocol of administrating the first dose with at least 10 vials at PHC/CHC level is being advocated to ensure that 90% of toxins are neutralized in every snakebite victim to ensure the saving of lives. Investigation of every death reported under the system or scanned in media reports using a standardized verbal autopsy form is a key tool adopted by the program addressing the issues responsible for snakebite deaths.

Moreover, upgrading emergency treatment facilities is one of the key priorities. Healthcare providers will undergo specialized and standardized training in snakebite treatment, and medical infrastructure will be enhanced to address the high-risk cases of Snakebite Envenoming.

Beyond improving clinical response, focus has also been placed on improving the transportation and referral system, as timely medical intervention is crucial in saving lives. Additionally, the program division has focused on conducting community awareness campaigns and educational programs, particularly targeting rural and tribal populations who are most vulnerable to snakebites. Some Information, Education, and Communication materials have also been developed. These initiatives will emphasize the importance of seeking immediate medical attention rather than relying on traditional remedies, which often exacerbate the situation. By dispelling myths and misconceptions, these programs will empower communities to respond effectively to snakebite emergencies.

To maximize the impact of these efforts, the MoHFW has taken significant steps to collaborate with stakeholders. Government agencies, healthcare institutions, non-governmental organizations (NGOs), and community organizations have been roped in a coordinated manner, pooling resources, expertise, and networks to achieve shared objectives. This collective approach will ensure that snakebite envenoming is tackled in a sustainable and effective manner.

Regular monitoring and evaluation mechanisms will also be implemented to assess progress, identify gaps, and make data-driven adjustments to strategies. By maintaining flexibility and responsiveness, the program can adapt to changing circumstances and emerging challenges over time.

Through this comprehensive and concerted effort, the ambitious goal of halving snakebite-related deaths by 2030 becomes both realistic and achievable. These initiatives will not only reduce the burden of snakebite envenoming but will also strengthen public health systems, build community resilience, and save countless lives, ultimately contributing to improved health outcomes and a healthier future for India.

## Figures and Tables

**Table 1:** Key Milestones in the Planning and Development of the National Action Plan for Prevention and Control of Snakebite Envenoming (NAPSE) Caption.

Month and Year	Key Milestones in the Planning and Development of the National Action Plan for Prevention and Control of Snakebite Envenoming (NAPSE)
March 2022	❖ In March 2022, a Regional Consultation on Snakebite Envenoming (SBE) in the WHO Southeast Asia Region (SEAR) revealed a critical gap. ⮚ Only 30% of SEAR countries had a dedicated national program, and just two countries had developed a national strategic plan for SBE prevention and control.
July 2022	❖ In response, India took a proactive step by organizing the First National Consultation on Developing NAPSE in July 2022, bringing together key stakeholders to initiate the process of formulating a national strategy. ❖ Based on the recommendations from this consultation, two major decisions were taken: ⮚ A proposal was approved in August 2022 to initiate a dedicated national program under the National Health Mission (NHM) to support States and Union Territories in undertaking snakebite prevention and control activities. ⮚ A multi-stakeholder core committee was constituted to develop NAPSE in November 2022, comprising representatives from: Ministry of Health and Family Welfare (MoHFW) Ministry of Environment, Forest and Climate Change (MoEFCC) Ministry of Fisheries, Animal Husbandry and Dairying (MoFAHD) Ministry of Panchayati Raj (MoPR) Ministry of Tribal Affairs (MoTA) NITI Aayog ICMR Other relevant stakeholders
February 2023	❖ The first meeting of the Core Committee was held on 28 February 2023, during which: ⮚ The first draft of NAPSE was shared for review. ⮚ The draft clearly defined the roles and responsibilities of each stakeholder, including Health, Wildlife, Animal Husbandry, Tribal Affairs, and others. ⮚ Each stakeholder reviewed their respective sections and suggested changes aligned with their mandates.
March 2023	❖ In March 2023, focused expert consultations were held with: ⮚ Clinicians and toxicologists ⮚ Public health researchers ⮚ Program nodal officers ⮚ Communication specialists ⮚ Civil society and NGO representatives These consultations helped refine the strategic components of NAPSE based on practical insights and field-level experience.
March–July 2023	❖ The revised draft was circulated multiple times to the Core Committee members for feedback and incorporation of sector-specific improvements.
August 2023	❖ In August 2023, a refined draft of NAPSE was presented at an inter-ministerial meeting with participation from senior officials of all stakeholder ministries. ⮚ Detailed discussions were held to define short-term, medium-term, and long-term actions for each ministry and department.
September 2023	❖ To ensure public participation and transparency, the draft NAPSE was also published on a public platform for broader feedback (comments were received from snakebite rescuers, snake survivors, private practitioners, global organizations, activists, etc.).
October 2023	❖ A landmark moment occurred in October 2023, when an inter-ministerial declaration was signed by the ministers of all key stakeholder ministries, signifying strong political will and high-level commitment to tackle the burden of SBE in India.
March 2024	❖ Official launch of the National Action Plan for Prevention and Control of Snakebite Envenoming (NAPSE) in India.

**Table 2:** Status of Snakebite Prevention Control Efforts in the Southeast Asia Region (Source: WHO-SERO).

Country	BAN	BTN	IND	INO	MDV	MMR	NPL	LKA	THA	TLS
National snakebite envenoming programs exist	No	No	Yes (2024)	No	No	Yes	Yes	No	Yes	No
Program in charge			COH NCDC, MoHFW			NCD program, MOH	Zoonotic and CD control, MOHP		Occupational disease	
National strategies or plans exist	Yes (2022–28)	No	Yes (2024)	No	No	No	No	No	Yes (2013)	No
National guidelines for treatment and management exist	Yes (2019)	Yes (2014)	Yes (2016)	Yes (2015)		Yes (2020)	Yes (2019)			

With the launch of NAPSE based on scientific findings of research studies across India, MoHFW has undertaken effective steps to address this issue in a multi-pronged manner. A summary of these activities is described in Table 3.

**Table 3:** Status of activities undertaken by the Centre for One Health, NCDC, and stakeholders for Snakebite Prevention and Control.

Gaps/Challenges Identified in Research Studies (Year 2000–2019)	Strategies Under NAPSE (Year 2022)	Activity Undertaken (Year 2021–2022 Onwards)	Action Under Progress
Venom-Related Issues—No Regional Venom Centers, exclusion of regional venoms in ASV, reliance on wild snakes, lack of quality testing, unregulated venom pricing, and no research on alternative ASV methods.	Ensuring the provision of anti-snake venom at all health facilities Institutionalization of regional venom centres Strengthening of the Poison Information Centre	Consultation with stakeholders on RVCs Standing committee for antisera formed for monitoring anti-snake venom-related safety and quality-related issues Identification of potential institutes for strengthening antisera	Guidelines for PICs and RVCs Operational research collaboration on alternative ASV methods. Collaboration for quality testing of antisera and venom
Surveillance and Legislative Gaps—Snakebite is not a notifiable disease, no standardized ICD coding, lack of structured education, and medico-legal complications delaying treatment.	Strengthening surveillance of human snake bite cases and deaths Notification of snakebite	Snakebite cases and deaths made as notifiable States to make it notifiable in respective public health acts or other available legal provisions Legal consultations on snakebite-related issues held by relevant ministries, e.g., MoEFCC * and Ministries of Law and Justice, on issues related to snake captivity, snake rescue, transport, etc. Human–Wildlife Conflict (HWC) guidelines on snakebite-related issues	Separate web portal on snakebite for ready information on snakebite-related activities Web portal with features for online notification of snakebite cases and deaths Zoo-win app developed for anti-snake venom (ASV) availability being piloted in five states Mobile app for snakebite notification and snake species information Collaboration with MoEFCC * for developing guidelines from the state’s forest department for snake rescuers
Medical and Diagnostic Challenges—Inadequate training, lack of venom detection kits, absence of standardized treatment protocols, no curriculum integration, missing life support training, no epidemiological study, and exclusion from government health schemes.	To strengthen the emergency care services at district hospitals/CHCs, including transport/referral services through 108 ambulance services Capacity building by training health professionals	Training in clinical and programmatic management undertaken for all states More than 2000 medical officers trained Standard protocol for initial management of snakebite prepared and disseminated PMJAY ** Govt. health insurance scheme covers snakebite	Airway and life support management training included in snakebite training Burden studies undertaken by ICMR Collaboration with stakeholders on diagnostic kit for identification of envenoming in snakebite victims
Public Awareness Deficiencies—Low awareness of snakebite prevention, lack of structured preventive measures, misinformation about ASV, and no dedicated 24/7 helpline.	Information Education and Communication (IEC) Public–Private Partnership Inter-sectoral Coordination	A standard IEC prototype with regional languages developed in the form of audio, video, booklet pamphlets etc. Funds provisioned for states/districts for IEC activity NGO mapping for collaboration undertaken and involved as NAPSE stakeholders Launch of Helpline in five states (Helpline number- 15400).	Central sector scheme proposal to cover NGO support for outreach activities Private sector collaboration for community awareness Helpline to be extended to all states/UTs
Research and Development Shortfalls—Need to focus on new ASV production technologies, alternative hosts for antibodies, or region-specific venom formulations.	Advocacy, support, and collaboration for research related to venom profiling Regional anti-venom Novel therapeutic for diagnosis and management of snake bite	ICMR projects underway on profiling of venom, preparation of anti-venom for northeastern states Snakebite burden assessment study by ICMR	Operational research issued and planned under the central sector scheme, e.g., dose-finding studies, venom profiling

* MoEFCC-Ministry of Environment Forest and Climate Change. ** Pradhan Mantri Jan Arogya Yojana (PM-JAY).

## Data Availability

No new data were created or analyzed in this study. Data sharing is not applicable to this article.
